# Ex post impact assessment of Marwa rehabilitation on irrigation performance under water scarcity in Egypt

**DOI:** 10.1038/s41598-025-34200-2

**Published:** 2026-01-20

**Authors:** Mohamed Embaby

**Affiliations:** 1 National Water Research Center (NWRC), Cairo, 13411 Egypt; 2 Water Management Research Institute (WMRI), National Water Research Center (NWRC), El-Qanater El-Khairia, 13621 Egypt

**Keywords:** Equitable water distribution, Irrigation efficiency, Land use optimization, Marwa rehabilitation, Smallholder farming, Water productivity, Environmental sciences, Hydrology

## Abstract

Egypt faces growing water scarcity due to population pressure and inefficient on-farm irrigation practices. Improving on-farm water-use efficiency is essential for sustaining agricultural productivity. This study assesses the long-term impacts of rehabilitating on-farm ditches (Marwas) serving 3–5 acre farms in the Hafez El Sharkeya Canal command area, Menia Governorate, five years after implementation. A combined technical and socio-economic assessment was conducted using hydraulic measurements, geometric surveys, and interviews with 40 farmers (20 beneficiaries and 20 non-beneficiaries). Results showed that rehabilitated Marwas with uniform, prismatic cross-sections facilitated better water conveyance and consistently met peak summer demand, while unimproved ones suffered from water losses and weed proliferation. Application efficiency for wheat increased from 50–60% in unimproved to 63–89% in rehabilitated Marwas. Water use efficiency increased from 0.59–0.69 in unimproved fields to 0.72–0.87 in rehabilitated systems. Water productivity for wheat was higher in rehabilitated Marwas (1.75 kg/m^3^) compared to unimproved ones (0.93 kg/m^3^). More than 80% of surveyed farmers preferred rehabilitated Marwas, citing reduced irrigation time, lower maintenance costs, and improved land utilization. The findings indicate that Marwas rehabilitation is associated with improvements in hydraulic performance, equity in water distribution, and greater farmer satisfaction with water delivery. The results offer relevant insights for efforts aimed at improving water-use performance in similar smallholder irrigation systems. However, broader multi-season and multi-location studies remain necessary before drawing generalized conclusions at the national scale.

**Supplementary Information:**

The online version contains supplementary material available at 10.1038/s41598-025-34200-2.

## Introduction

Egypt is confronting an intensifying water scarcity challenge, driven by its arid climate, limited freshwater resources, rapid population growth, and the growing impacts of climate change^1^. The Nile River supplies 94% of the country’s renewable freshwater resources [2, and per capita availability is projected to decline below 446 m^3^/capita in 2037, far beneath the global water scarcity threshold of 1000 m^3^/year^3^. By 2030, water demand in Egypt’s agricultural sector could exceed supply by 20–25%, posing risks to irrigation performance, food security, and rural livelihoods^2^.

Given this situation, improving irrigation efficiency and water distribution has become a national priority, as the agricultural sector consumes more than 80% of Egypt’s total water resources^4–6^. In response, the Egyptian government has launched modernization programs, including laser land levelling, gated pipe systems, and canal lining, to optimize water distribution and reduce conveyance losses^7,8^. These interventions aim to minimize water losses, enhance delivery reliability, reduce operational losses, and promote equitable distribution (water allocation). However, their long-term success depends on sustained maintenance, participatory management, and effective institutional frameworks^9^.

Egypt’s irrigation network spans approximately 45,000 km, serving nearly 8 million feddans nationwide. It consists of a hierarchical structure of main, secondary, and tertiary canals, extending down to on-farm ditches known as Marwas, which deliver water by gravity to individual fields. However, traditional surface irrigation methods, predominantly free-flow and flood irrigation, remain widely used among farmers. These practices often result in low application efficiency, high conveyance losses, and significant inequitable among farmers^10^.

The Ministry of Water Resources and Irrigation (MWRI) has launched a strategic plan that includes lining distributary canals to boost water transmission and distribution efficiency. However, the program’s on-farm outcomes and long-term effectiveness remain poorly understood. Evaluating these impacts is crucial to guide future investments and ensure water equity among farmers, aligning with Egypt’s National Water Resources Plan (2037) and the UN Sustainable Development Goal 6^11,12^.

Canal lining can reduce seepage losses by up to 0.2 million m^3^ per kilometer annually, especially in sandy soils^4,12–14^. However, these benefits are limited in clayey soils due to naturally low permeability. Seepage is never fully eliminated, and without regular maintenance, canal linings may degrade over time, reducing efficiency^1,15–20^. Moreover, most evaluations have focused on physical performance rather than integrated socio-economic and environmental outcomes, leaving uncertainty about the actual long-term effectiveness.

While numerous studies have examined rehabilitation at main and distributary canal levels^4–6,10,11,15,20–22^, far fewer have investigated tertiary or on-farm canals (Marwas), where inequities and operational challenges are most pronounced. Research has primarily emphasized hydraulic performance or short-term improvements, often neglecting water distribution equity, crop-level water use efficiency, and farmer acceptance. This gap limits understanding of how tertiary canal rehabilitation affects efficiency, equity, and local adoption.

Given the limited availability of comprehensive assessments at the on-farm scale, this study presents an ex-post evaluation of rehabilitated tertiary canals (Marwas) within the command area of the Hafez El Sharkeya Canal in Menia governorate, Upper Egypt. It applies a mixed-methods framework that integrates field measurements, irrigation performance indicators, and participatory farmer surveys. The findings aim to guide irrigation modernization, investment prioritization, and participatory water management for sustainable water use and equitable agricultural development. The specific objectives of ex-post assessment are to:

- Assess the hydraulic performance and operational convenience of rehabilitated Marwas;
- Evaluate irrigation adequacy relative to crop water requirements and consumption;
- Quantify water savings and improvements in application efficiency and water use-efficiency;
- Compare performance indicators with unimproved (control) Marwas;
- Examine socio-economic impacts on farmers’ livelihoods and rural incomes following on-farm improvements.

## Materials and methods

### Study area

**Fig. 1:**
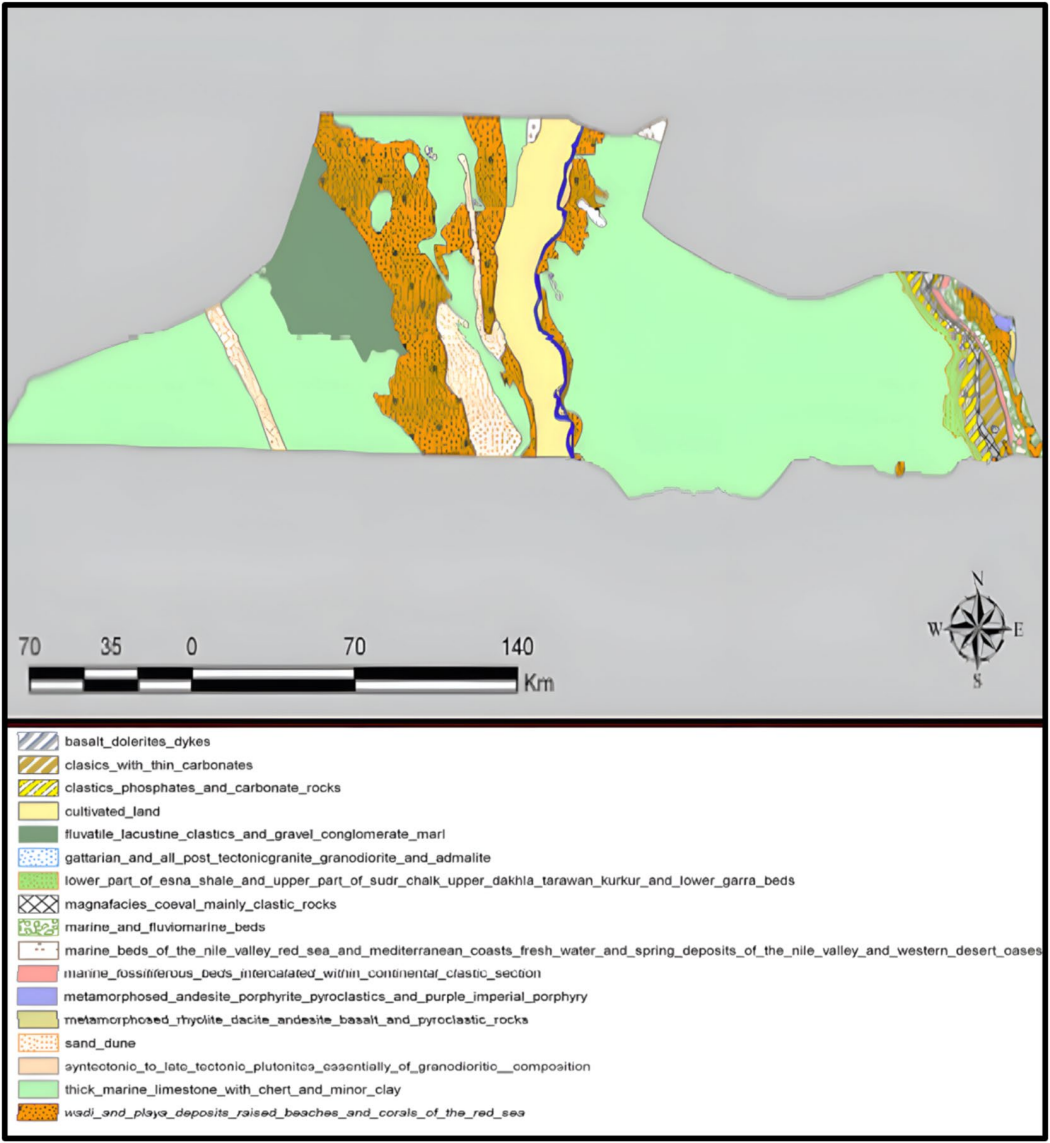
Soil classification map of Menia governorate. Map generated using ArcGIS Desktop 10.8 (Esri, Redlands, CA, USA; https://www.esri.com/en-us/esri-press/browse/getting-to-know-arcgis-desktop-10-8).

The study was conducted in Menia Governorate in northern Upper Egypt. Menia contains approximately 2262 km^2^ of cultivated land by Beni Suef to the north and Giza to the west^23^. The governorate has a continental climate influenced by the north-northwest winds. Its location on both sides of the Nile helps moderate temperature extremes relative to interior desert areas. Most soils in Menia are non-saline and fertile, making them suitable for agriculture (Fig. 1). Three main land types occur in the governorate: (1) Modern alluvial lands, formed by recent Nile sedimentation (~ 70% of cultivated area), mostly located between the Nile River and the Sea of Joseph (Ibrahimiya Canal) in the west, (2) Old alluvial lands (aeolian) along the western edge of the cultivated Nile valley, (3) Surface calcareous lands on eastern slopes washed from the plateau.

**Fig. 2:**
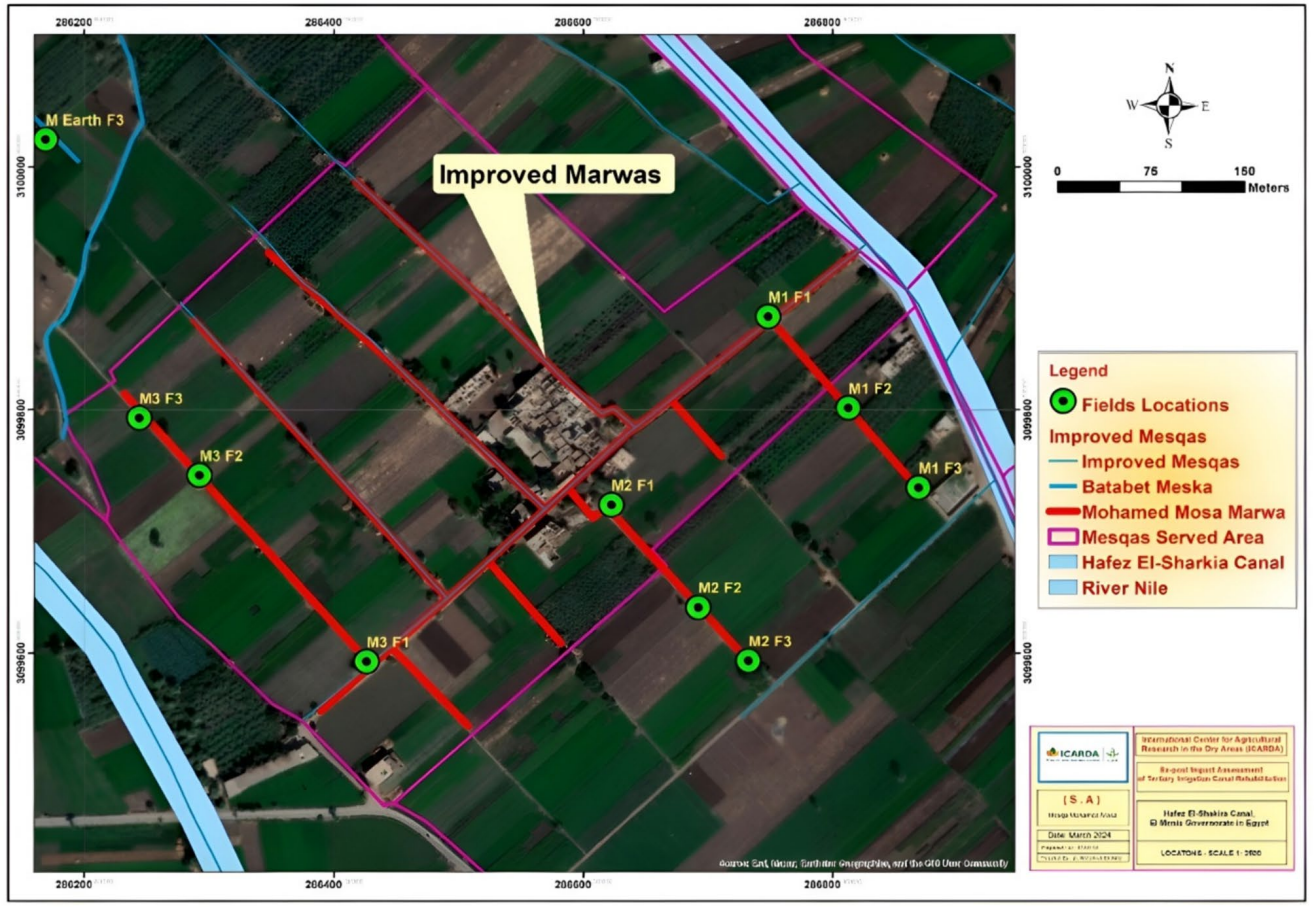
Distribution of the improved Marwas and associated cultivated fields within the Mohamed Mousa Mesqa Command area, Menia Governorate, Egypt. Map generated using ArcGIS Desktop 10.8 (Esri, Redlands, CA, USA; https://www.esri.com/en-us/esri-press/browse/getting-to-know-arcgis-desktop-10-8).

**Fig. 3:**
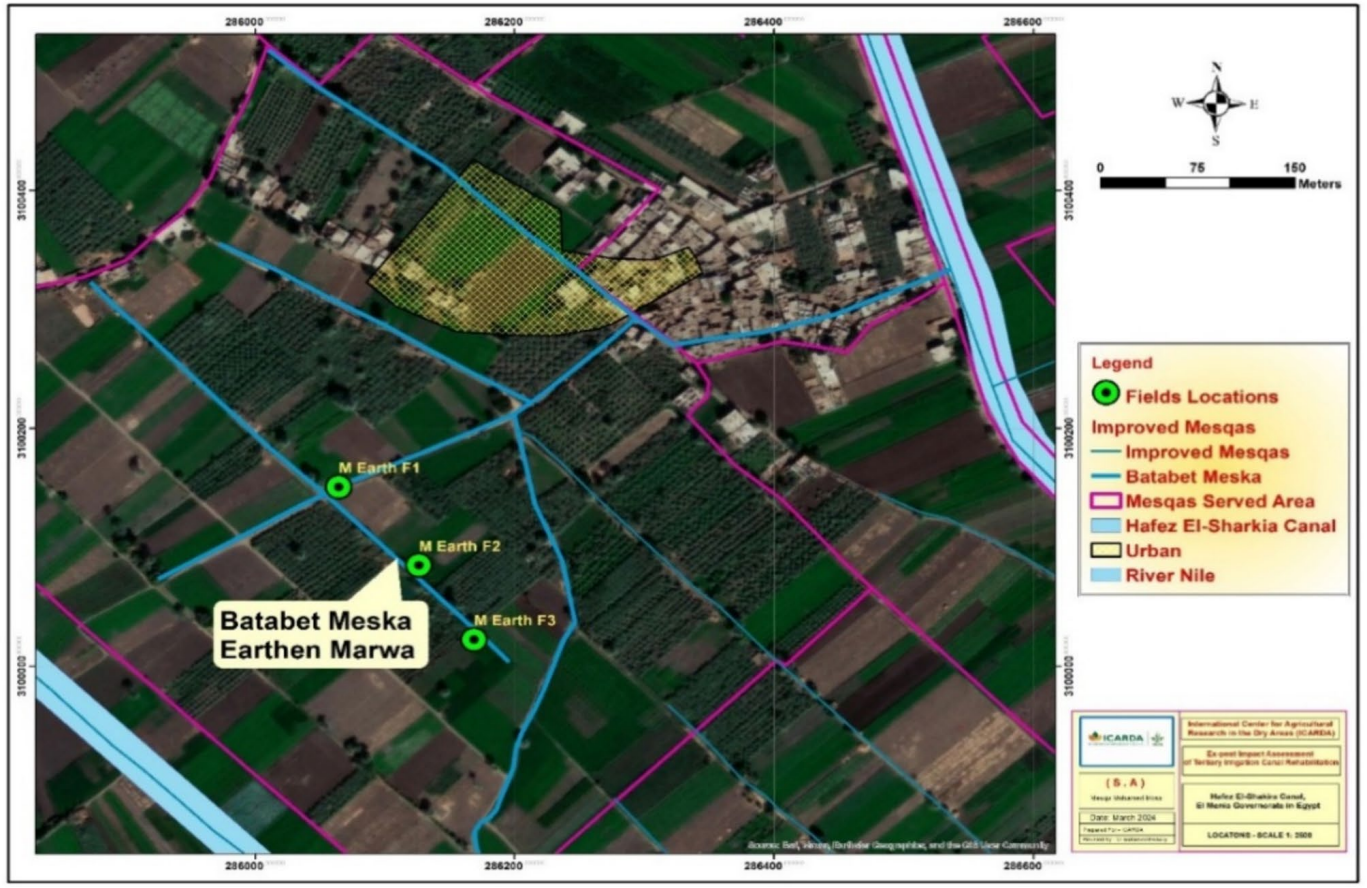
Spatial distribution of field sampling points along the improved Marwas (M1, M2, & M3) and the unimproved control Marwa (M4). Field codes denote sampling locations along each Marwa: start (F1), middle (F2), and end (F3). Map generated using ArcGIS Desktop 10.8 (Esri, Redlands, CA, USA; https://www.esri.com/en-us/esri-press/browse/getting-to-know-arcgis-desktop-10-8).

**Fig. 4:**
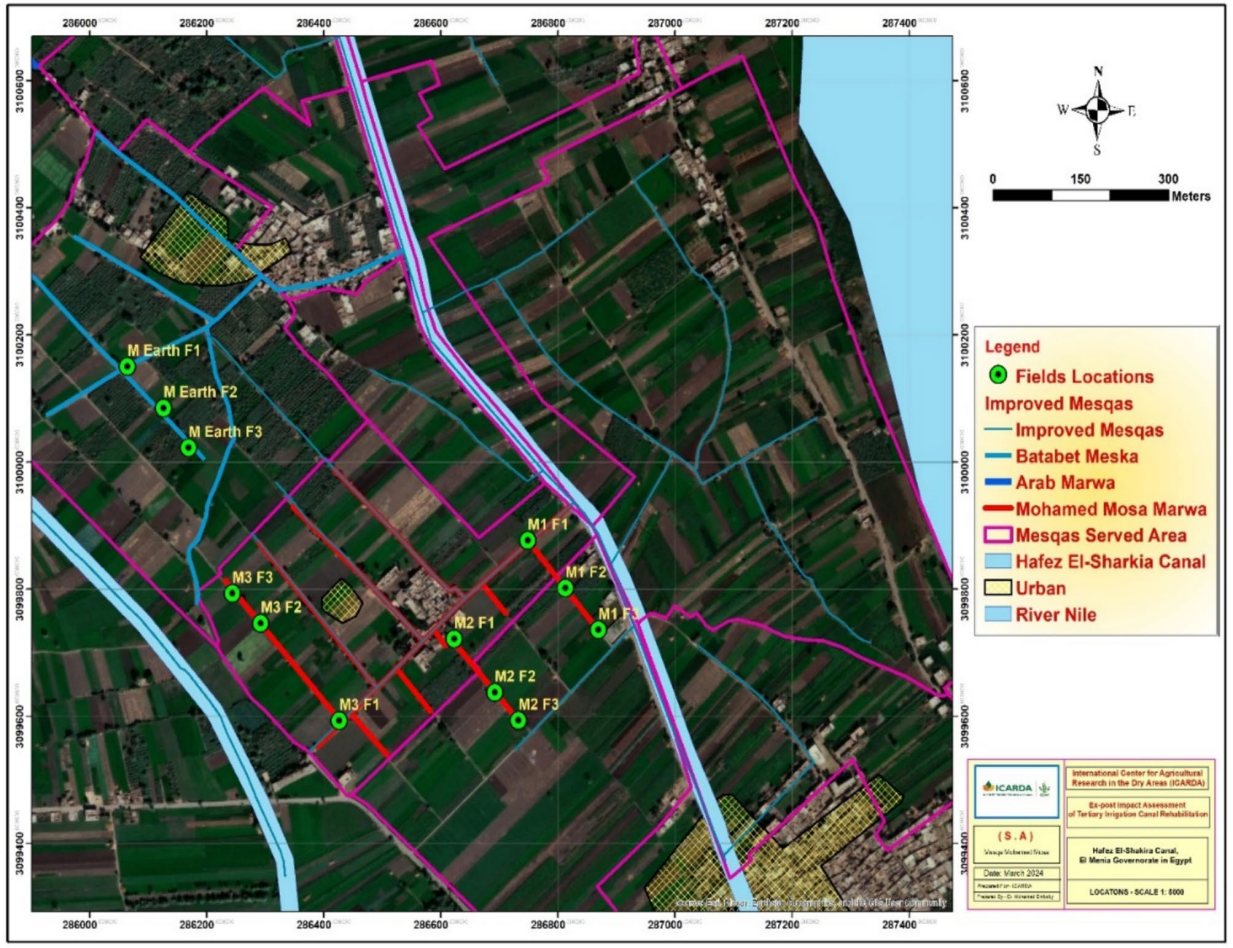
Location of the unimproved (earthen) Marwa under the Batabet Mesqa system. Map generated using ArcGIS Desktop 10.8 (Esri, Redlands, CA, USA; https://www.esri.com/en-us/esri-press/browse/getting-to-know-arcgis-desktop-10-8).

The study focused on the Hafez El-Sharkeya Canal command area (27.7 km, serving ~ 8380 acres). Two tertiary canal systems (Mesqas) were selected for comparison: (1) Mohamed Mousa Mesqa (lined/improved (rehabilitated), 2.2 km, ~ 70 feddans), and (2) Batabet Mesqa (unimproved earthen control, 1.3 km, ~ 46 feddans). Three rehabilitated Marwas (head, middle, tail reaches) and three control Marwas were monitored. Each Marwa included three representative farms, totaling 18 sample farms (9 improved + 9 controls). Farms were selected using purposive stratified sampling, ensuring coverage of upstream–midstream–downstream reaches, dominant crops (wheat, clover, sugar beet, taro), and varying field sizes, Table 1. All farms were geo-referenced using Trim-GPS units’ geo-referenced farms and Marwas, with spatial layers were processed in Arc-GIS 10.8 and mapped (Figs. 2, 3, 4).

**Table 1:** Characteristics of sampled farms, including field area, coordinates, and cropping patterns.

Code	Area acres	Latitude mE	Longitude mN	Crops
M2 F2	18	3,099,637.49	286,692.01	Sugar beet
M2 F1	18	3,099,721.87	286,622.46	Clover
M2 F3	18	3,099,593.67	286,732.23	Wheat
M Control F1	16	3,100,150.6	286,064.37	Wheat, Figs
M Control F2	18	3,100,084.83	286,126.05	Wheat
M Control F3	18	3,100,022.49	286,168.67	Wheat, Figs
M1 F3	20	3,099,735.97	286,868.68	Clover
M1 F2	20	3,099,801.63	286,812.45	Wheat, Taro
M1 F1	20	3,099,876.68	286,748.19	Wheat, Clover
M3 F3	1	3,099,793.28	286,243.84	Wheat
M3 F2	18	3,099,746.23	286,292.19	Clover
M3 F1	1	3,099,592.95	286,426.12	Sugar beet

Figures 2, 3, 4 illustrate the locations of the selected improved (M1, M2, and M3) and unimproved (M4) Marwas and their corresponding fields farms, while Table 1 summarize the field characteristics, including served area, cropping patterns, and geographic coordinates.

### Methodology overview

All procedures followed relevant guidelines and regulations and were approved by the National Water Research Center (NWRC) Research Ethics Committee. Informed consent was obtained from all subjects involved in the study.

Fieldwork and monitoring were conducted during the winter irrigation season 2023/2024. The study applied a comparative ex-post design to evaluate the technical, environmental, and socio-economic impacts of tertiary canal rehabilitation. This mixed-methods framework integrates hydraulic measurements, soil moisture dynamics, and farmer perceptions to capture both quantitative performance and user experience. Key performance metrics were derived from both field measurements and stakeholder input, highlighting the potential benefits and limitations of Marwa lining and rehabilitation. The assessment consisted of three main components:

- Field data collection from soil and irrigation systems.
- Analytical assessment of performance indicators.
- Socio-economic evaluation through farmer engagement and surveys.

#### Field data collection

##### Measurement of applied water and Marwa discharge

**Fig. 5:**
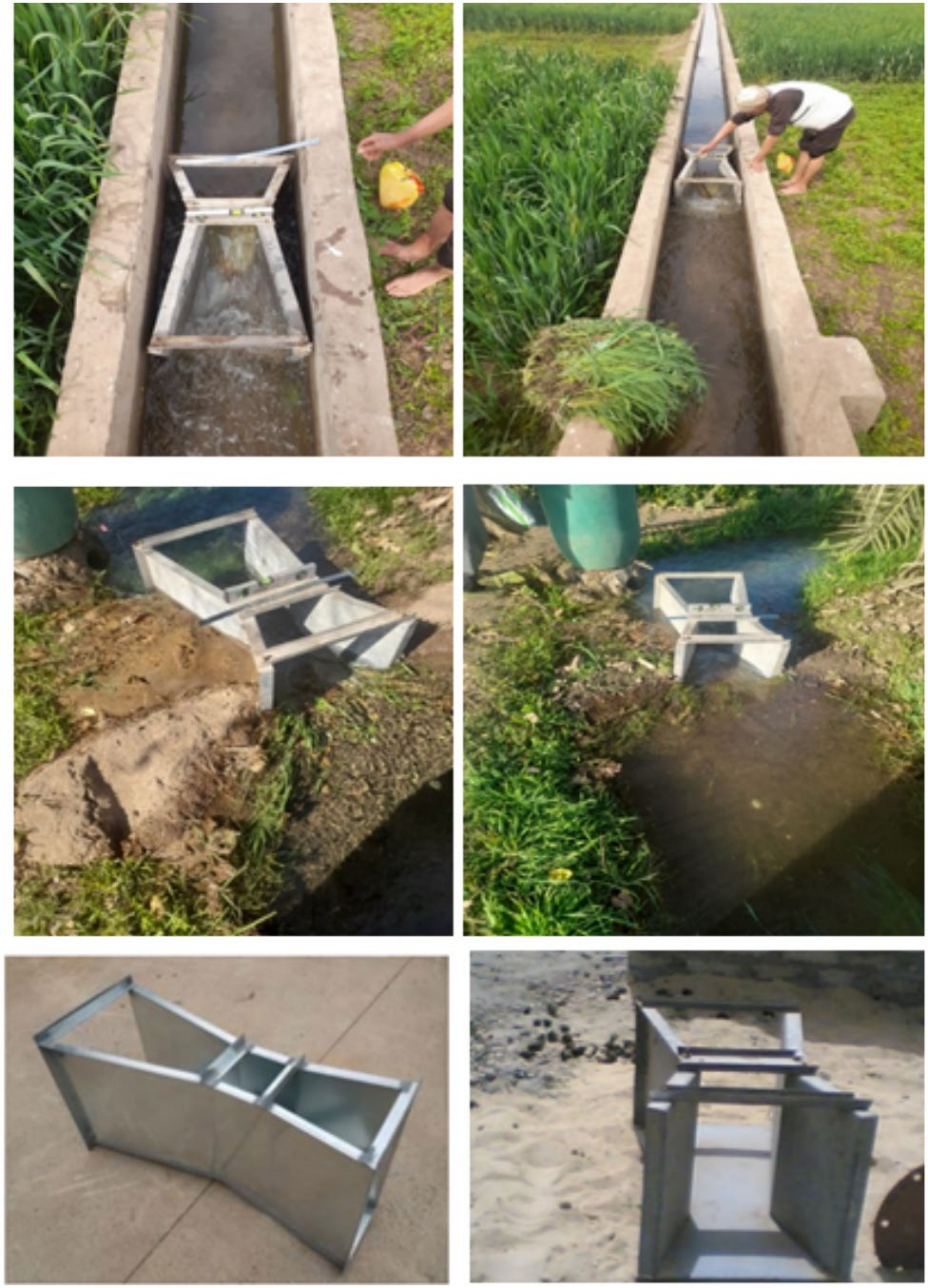
Parshall flume for flow measurements in the selected Marwas.

**Fig. 6:**
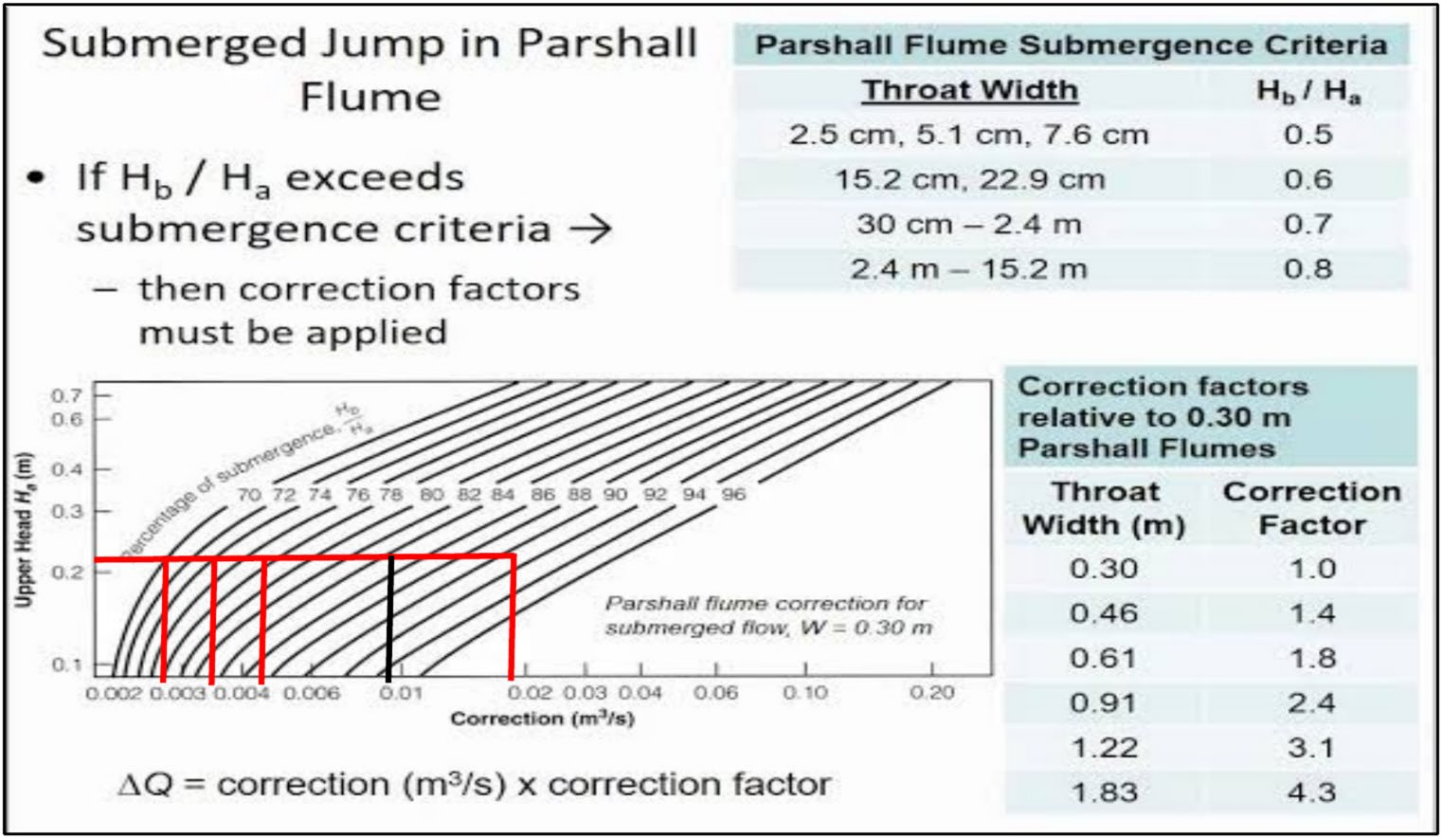
Main evaluation equation, calibration constants, submergence correction, and setting up of Parshall Flume.

Flow discharge was measured using a Parshall flume (Fig. 5), installed under operational conditions, Table 2. The flume was used in this study to measure volumetric flow rate in irrigation discharges (Fig. 5). Parshall flumes measured flow rates at Marwa inlets and outlets with submergence corrections applied. Calibration followed manufacturer specifications (ASTM D1941 standards), and measurement uncertainty (estimated within ± 3%) was minimized by repeating each measurement three times per event (Figs. 5, 6). The free-flow discharge rate (Q) can be summarized in this equation^24^:

1 $$\mathrm{Q}= {\mathrm{CH}}^{\mathrm{n}}$$

where, Q = flow rate, C = free-flow coefficient for the flume (Table 2), H = head at the primary point of measurement, & n varies with flume size (Table 2).

**Table 2:** Free-flow coefficients (C) and exponents (n) used for discharge calculation in Parshall flume.

Throat width	Coefficient (C)	Exponent (n)
1 in	0.338	1.55
6 in	2.08	1.58
2 ft	8	1.55
6 ft	24	1.59
10 ft	39.38	1.60
20 ft	76.25	1.60
30 ft	113.13	1.60
40 ft	150	1.60
50 ft	186.88	1.60

##### Soil sampling and analysis

Soil Eijkelkamp augers were employed to collect disturbed soil samples from three depths (0–30 cm, 30–60 cm, and 60–90 cm) at head, middle, and tail farms of each Marwa, Table 3. Sampling occurred 48 h before and after irrigation, yielding a total of 72 samples (36 from improved Marwas, & 36 from controls) to capture short-term changes in soil moisture and storage at three depths. Each farm contributed six samples (3 depths × 2 time points = 6 samples) to capture within-field variability. To minimize spatial bias, each field was divided into quadrants, and samples were taken diagonally across plots. Table 3 summarizes sampling locations along the Mohamed Mousa Mesqa, depth, and crops.

**Table 3:** Soil samples locations, depths, and crop types from improved and unimproved Marwas.

Marwa no	Location	Field code	Sampling depths (cm)	Crop(s)
M1	Start	M1F1	30, 60, 90	Clover & Wheat
	Middle	M1F2	30, 60, 90	Wheat & Taro
	End	M1F3	30, 60, 90	Clover
M2	Start	M2F1	30, 60, 90	Clover
	Middle	M2F2	30, 60, 90	Sugar Beet
	End	M2F3	30, 60, 90	Wheat
M3	Start	M3F1	30, 60, 90	Sugar Beet & Clover
	Middle	M3F2	30, 60, 90	Clover
	End	M3F3	30, 60, 90	Wheat
M4 (Control)	Start	McontrolF1	30, 60, 90	Clover
	Middle	McontrolF2	30, 60, 90	Clover
	End	McontrolF3	30, 60, 90	Wheat

Samples were transported to the laboratory and oven-dried at 105 °C for 24 h following standard procedures (WiseVen WON Standard Oven)^25,26^ to determine gravimetric moisture content. QA/QC procedures included duplicate drying of 10% of samples, blanks, and balance calibration. were applied. All sample locations were geo-referenced with a handheld GPS (accuracy ± 3–5 m) and entered into a GIS database.

##### Farmers’ questionnaire

A structured questionnaire was designed to capture farmers’ perceptions of irrigation performance, costs, and benefits. Data were collected from a sample representing 20% farmers from each canal reach (head, middle, and tail) within the command area. This included 20 beneficiaries (those with access to improved Marwas) and 20 non-beneficiaries (those using traditional earthen Marwas) from an estimated total population of 180 farmers across both canal systems. Although the sample size is modest, the stratified selection ensured proportional representation across canal reaches and farm sizes, and crop types, thereby meeting the requirements for exploratory socio-technical assessments where variability and perception heterogeneity are more critical than population size alone. The questionnaire was originally developed in Arabic to ensure clarity and accessibility for local farmers, while the English version is provided in Appendix I for reference. To minimize interviewer bias, two trained facilitators from the local Water Users Association (WUA) conducted the surveys using a standardized script, with responses verified through follow-up visits. The questionnaire addressed six aspects:

- Time and money saving associated with irrigation practices
- Water quality enhancement perceived by farmers
- Canal weeds are absence due to lined structures
- Water control and regulation at the field level
- Cultivation of new or higher-value crops
- Reducing effort and increasing irrigation efficiency

This mixed-methods approach combines quantitative indicators and qualitative perceptions, enabling a comprehensive evaluation of the Marwa rehabilitation program’s effectiveness in both technical and socio-economic terms. It also aligns with best-practice guidelines for participatory irrigation assessment proposed by van Halsema & Vincent^27^, emphasizing user-centered eater management and farmer engagement as key drivers of sustainable performance improvement.

#### Data analysis for assessment

##### Marwa geometric assessment

A comprehensive topographic survey was conducted to generate schematic diagrams representing the cross-sectional and longitudinal profiles of selected Mesqas and Marwas. These profiles were documented at control points along the sampled irrigation channels. The geometric data obtained were used to evaluate the current as-built conditions, including the structural dimensions and water conveyance capacities, of both rehabilitated and traditional earthen channels. This assessment facilitated the verification of the actual capacity of the Marwas against their intended design specifications.

##### Performance indicators

Performance included application efficiency (Ea), water use efficiency (WUE), water use index (WUI), water productivity (WP), equity, and dependability.

Water application efficiency (Ea)—fraction of water quantity stored in the root zone to the quantity of water applied to the field^28^. Thus,

2 $$\mathrm{Ea}=\left(\frac{{\mathrm{W}}_{\mathrm{s}}}{{\mathrm{W}}_{\mathrm{a}}}\right)\times 100$$

where Ws = water stored in the root zone (m^3^) and Wa = water applied to the field (m^3^). Storage in the root zone was computed from measured pre- and post-irrigation soil moisture (volume basis) integrated over the sampling depth.

Water use efficiency (WUE) calculated as the ratio of yield (kg) to the total volume of applied irrigation water (m^3^)^29,30^. Thus,

3 $$\mathrm{WUE}= \frac{\mathrm{Yield}}{\text{Applied water }({m}^{3})}$$

Water use index (WUI)—The ratio between the applied water and actual requirements^31^. Thus,

4 $$\mathrm{WUI}= \frac{\text{Applied water}}{\text{Crop water requirements}}$$

Water productivity (WP)—Crop production (yield) per unit amount of water (kg/m^3^ or ton/m^3^) used. The crop’s yield could be expressed as the weight of the crop, its seeds, or its grains. Based on the framework by Zwart and Bastiaanssen^30^. Thus,

5 $$\mathrm{WP}= \frac{\text{Seeds yield }(\frac{\mathrm{ton}}{\mathrm{feddan}})}{\text{Applied water }(\frac{{\mathrm{m}}^{3}}{\mathrm{Feddan}})}$$

Equity and dependability indicators were assessed through structured farmer surveys aimed at capturing users’ experiences and perceptions of the irrigation system. Equity refers to the fairness and uniformity of water distribution across different users and locations, ensuring that no farmer is disadvantaged in terms of water access. Dependability, on the other hand, reflects the system’s consistency and reliability in delivering water under various conditions, including peak demand periods and environmental stress. Together, these indicators offer valuable insights into the operational integrity of the Marwa system, helping to identify both strengths and areas requiring further enhancement. This qualitative data provided insights into the social performance of the system, in line with Meinzen-Dick^32^.

#### Statistical analysis

To ensure a comprehensive evaluation of the performance of improved versus traditional Marwas, statistical analysis was integrated with both qualitative and quantitative diagnostic tools in a unified framework. The analysis included the following components:

- A one-way ANOVA: A one-way Analysis of Variance (ANOVA) was applied to assess differences between beneficiaries and non-beneficiaries regarding farmers’ perceptions of irrigation performance, operational costs, and benefits. ANOVA was chosen because it provides a robust statistical framework for comparing mean responses between independent groups, ensuring that observed differences were statistically significant rather than due to random variation. The analysis was conducted at a 95% confidence level (*p* < 0.05).
- Sensitivity analysis: To evaluate the robustness of technical indicators, including application efficiency (Ea), water use efficiency (WUE), and water use index (WUI), a ± 5% variation was applied to the measured discharge and soil-moisture values. This range accounted for measurement uncertainty (± 3%) and natural field variability. The analysis confirmed that the calculated indicators remained stable and reliable within this range.
- Pareto analysis: A Pareto chart was used to identify and prioritize the key factors influencing farmers’ adoption of the Marwa improvement system. The analysis highlighted the most frequent reasons, including absence of weeds, ease of irrigation, time and cost savings, and improved water quality, which together accounted for the majority of adoption motivation.
- Ishikawa (Fishbone) diagram: To understand the factors influencing farmers’ acceptance or rejection of Marwa rehabilitation, an Ishikawa (cause-and-effect or fishbone chart) diagram was employed^33,34^. This analytical tool organizes and visualizes the root causes affecting the effectiveness of the Marwa improvement system, helping to compare the relative importance of different factors.
- Contributing factors were categorized into six main groups: Measurements, Material, Personnel, Machines, Methods, and Environment. (1) Measurement factors included crop productivity, distribution equity, and application efficiency, while (2) material factors involved pump efficiency and irrigation cost. (3) Personnel factors, such as land leveling, maintenance, weed control, and conflict management, and (4) Machine-related aspects, like pumps and leveling technologies, reflected technical requirements. (5) Method factors, including rotation time, Marwa length, and weed presence, addressed operational practices, whereas (6) Environmental factors captured pollution reduction and sustainability impacts. These factors were further classified as: (1) controllable variables, which can be directly managed or adjusted (such as ease of maintenance and fuel availability); (3) intermediate variables, which can be influenced but are not fully controllable (like irrigation scheduling); and (4) uncontrollable (bothering) variables, which lie outside the scope of intervention and cannot be modified or controlled (such as the physical location of a field along the Marwa). A cause-to-result diagram was then applied to identify critical factors, clarifying the interactions among technical, managerial, and environmental elements that shape the success of the Marwa rehabilitation.
- Integrated performance evaluation framework (IPEF): Within the IPEF framework, tertiary canal performance was evaluated by integrating hydraulic measurements with socio-economic surveys. Key indicators (conveyance efficiency, application efficiency, and water productivity) were compared using ANOVA (SPSS v28) at a 95% confidence level to assess differences between improved and unimproved canals. Farmer data were analyzed descriptively and correlated with technical metrics to examine links between infrastructure improvements and satisfaction, participation, and perceived equity.

## Results and discussion

Rehabilitation of tertiary irrigation channels (Marwas) significantly enhanced hydraulic performance, reduced friction losses, and improved water distribution equity. In contrast, unimproved earthen Marwas exhibited irregular, non-uniform cross-sections, high roughness, and excessive weed growth, leading to substantial conveyance losses and localized waterlogging, particularly in low-lying areas.

### Evaluation of Marwas’ conveyance capacity

The geometric survey of both improved and unimproved Marwas revealed major differences in cross-section uniformity, bed slope stability, and hydraulic roughness. Rehabilitated Marwas demonstrated more regular, prismatic, and uniform longitudinal and uniform cross-sectional shapes with consistent slopes, directly contributing to lower Manning’s roughness coefficients (n = 0.014–0.018). These improvements facilitated smoother water flow, enhanced hydraulic performance, reduced friction losses, and improved equity in water distribution^29,35,36^. These characteristics are known to support consistent flow velocities and minimize stagnation zones that typically encourage weed proliferation and waterlogging. These results are consistent with Ashour et al.^37^, who emphasized that well-designed and properly constructed canal lining markedly enhances irrigation efficiency, whereas poor execution can lead to structural deterioration. They are also consistent with Elkamhawy et al.^38^, who reported that lining irrigation canals in the Nile system reduced seepage losses and increased conveyance efficiency up to 99% compared to about 25% in unlined sections. Overall, the present findings illustrate that effective rehabilitation is essential for sustainable tertiary canal performance.

In contrast, the unimproved earthen Marwas displayed irregular, non-prismatic sections and inconsistent slopes, leading to high Manning’s roughness coefficients (n = 0.035–0.045), significant conveyance losses, and overall poor hydraulic performance^39^. These losses were exacerbated by excessive aquatic weed growth due to low velocities, especially during off-peak irrigation periods, which further reducing field-level water availability. The non-uniformity also caused inequitable water distribution, where head-end farmers received sufficient water, while tail-end users often experienced shortages.

**Fig. 7:**
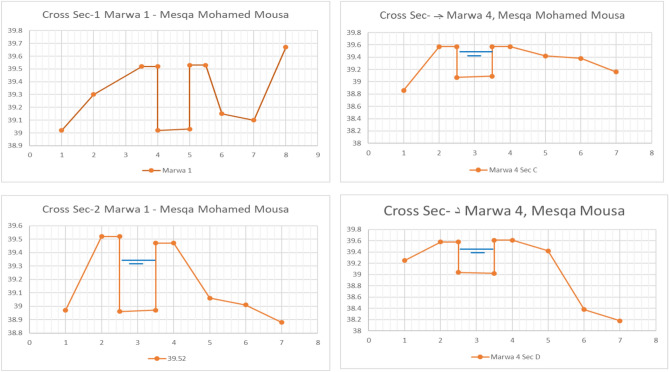
Cross-sections of improved Marwas (M1–M4) along Mohamed Mousa Mesqa.

**Fig. 8:**
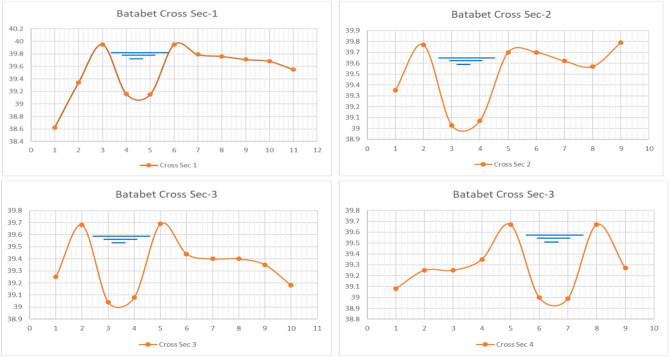
Cross-sections of the unimproved earthen Marwas (Sects. 1-4)"Introduction–Conclusion") under the Batabet Mesqa system.

**Fig. 9:**
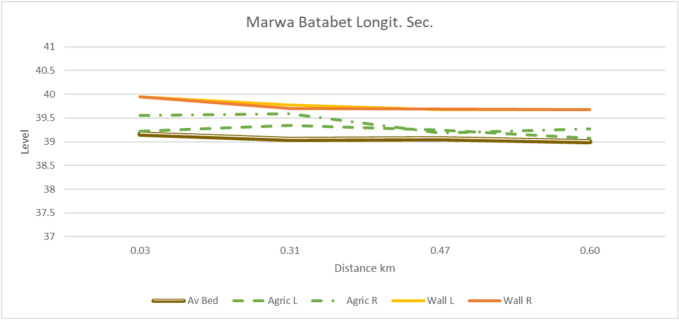
Longitudinal section of Marwa Batabet, showing the average bed level (Av. Bed), agricultural left and right banks (Agric. L/R), and canal wall boundaries (Wall L/R).

Figures 7, 8, and 9 clearly illustrate these differences. Cross-sections of three improved Marwa samples along the Mohamed Mousa Mesqa (head, middle, and tail) retained their design dimensions and alignment, confirming the structural stability and effectiveness of rehabilitation measures. Conversely, earthen Marwas displayed notable silt deposition, lateral seepage zones, and degraded banks, all of which contributed to reduced conveyance due to increased friction losses. Furthermore, the low water velocities in these channels facilitated the growth of aquatic weeds, particularly in stagnant zones. Hydraulic reassessment of as-built conditions confirmed that the improved Marwas were capable of meeting irrigation demands during July and August, aligned with the local cropping pattern.

### Soil moisture content

Tables 4, 5, and 6 present pre- and post-irrigation soil moisture content (MC) measured at depths 0–30, 30–60, and 60–90 cm. The results demonstrate enhanced soil moisture retention and better infiltration uniformity under improved Marwas compared to unimproved systems. In the improved Marwa field M3F1, the average soil moisture increased from 11.41 to 12.79 cm (≈12.79% gain) after irrigation, reflecting effective infiltration and retention within the root zone. Similarly, M3F3 showed uniform increases across depths (≈5%), confirming efficient vertical percolation and minimal surface runoff^40^.

**Table 4:** Pre- and post-irrigation soil moisture at three depths in field M3F1.

	Before irrigation				48 h after irrigation			
Location A	Depth	MC (m)	MC (cm)	Mean ± SD	Depth	MC (m)	MC (cm)	Mean ± SD
	cm				cm			
	30	0.1023	10.23	11.41 ± 1.03	30	0.1325	13.25	12.79 ± 0.40
	60	0.1211	12.11		60	0.12522	12.52	
	90	0.1188	11.884		90	0.1259	12.59

**Table 5:** Pre- and post-irrigation soil moisture at three depths in field M3F3.

	Before irrigation				48 h after irrigation			
Location A	Depth	MC (m)	MC (cm)	Mean ± SD	Depth	MC (m)	MC (cm)	Mean ± SD
	cm				cm			
	30	0.1240	12.40	12.66 ± 0.57	30	0.1349	13.49	13.34 ± 0.31
	60	0.1227	12.27		60	0.1355	13.55	
	90	0.1332	13.32		90	0.1298	12.98

**Table 6:** Pre- and post-irrigation soil moisture at three depths in field M3F2.

	Before irrigation				48 h after irrigation			
Location A	Depth	MC (m)	MC (cm)	Mean ± SD	Depth	MC (m)	MC (cm)	Mean ± SD
	cm				cm			
	30	0.1278	12.78	10.51 ± 3.54	30	0.1414	14.14	11.60 ± 2.20
	60	0.0644	6.44		60	0.1023	10.23	
	90	0.1232	12.32		90	0.1042	10.42	

In contrast, M3F2 (unimproved) displayed a heterogeneous moisture pattern, with limited increases across all depths. The surface layer showed the most variability, rising only from 10.51 ± 3.54 to 11.60 ± 2.20 cm, while deeper layers (60–90 cm) displayed weak gain. These inconsistent moisture responses indicate restricted infiltration, likely due to soil compaction, uneven water application, or factors commonly associated with unimproved or poorly maintained Marwas^41^. Such variability could lead to suboptimal root zone moisture and reduce overall irrigation efficiency.

These patterns are consistent with Doorenbos and Pruitt^42^ highlighted that lined or rehabilitated systems improve infiltration uniformity by stabilizing flow velocity and reducing seepage-induced heterogeneity. Moreover, Hillel^43^ and Seyfried et al.^44^ (2004) demonstrated that soil compaction and stratification in traditional irrigation systems lead to irregular infiltration and low field efficiency. The results underscore that canal rehabilitation indirectly enhances soil–water interaction by stabilizing delivery rates and maintaining consistent infiltration conditions. This improvement forms the basis for higher water application efficiency and water use efficiency observed later in Sect. “Obtained performance indicators”.

### Cropping pattern and crop consumption

Crop water consumption (ETc) was determined using crop-specific coefficients (Kc) and FAO Penman–Monteith-based reference evapotranspiration (ETo) values for the winter season, to evaluate whether the available irrigation supply from the improved and unimproved Marwas met the water requirements of local crops. Seasonal ETc values (Table 7) ranged from 299 mm for faba bean to 948 mm for sugar cane, reflecting the diversity of crop water demands across Menia’s farming systems. High-demand crops such as sugar cane and cotton recorded seasonal ETc values of 948.79 mm and 873.21 mm, respectively, whereas wheat and faba bean required only 396.68 mm and 299.05 mm. This differentiation underscores the necessity of precise irrigation delivery and scheduling to align supply with crop-specific demands.

**Table 7:** Cultivation calendar, crop coefficients (Kc) for different growth stages, and seasonal ETc for main crops in Menia governorate^45^.

Crop	Plant date	Harvest date	Growth stages	Kc/stage	Seasonal ETc (mm)
Wheat	1-Nov	20-Apr	1-Nov, 5-Dec, 16-Jan, 9-Mar, 20-Apr	0.32, 0.32, 1.10, 1.10, 0.55	396.68
Faba Bean	1-Nov	30-Mar	1-Nov, 4-Dec, 24-Jan, 14-Mar, 30-Mar	0.32, 0.32, 1.00, 1.00, 0.85	299.05
Barley	15-Nov	9-Apr	15-Nov, 14-Dec, 19-Jan, 4-Mar, 9-Apr	0.22, 0.22, 1.10, 1.10, 0.55	315.89
Clover	1-Nov	10-May	1-Nov, 18-Dec, 4-Feb, 24-Mar, 10-May	0.57, 0.57, 1.15, 1.15, 0.80	505.64
Onion	1-Sep	1-Mar	1-Sep, 19-Sep, 18-Oct, 15-Jan, 1-Mar	0.35, 0.35, 1.20, 1.20, 0.55	532.39
Sugar Beet	15-Nov	15-May	15-Nov, 12-Dec, 4-Feb, 9-Apr, 15-May	0.35, 0.35, 1.15, 1.15, 0.95	568.38
Cotton	15-Feb	24-Aug	15-Feb, 15-Mar, 3-Apr, 27-Jul, 25-Aug	0.31, 0.31, 0.95, 0.95, 0.50	873.21
Sugar Cane	1-Feb	31-Jan	1-Feb, 1-Feb, 4-Apr, 7-Oct, 31-Jan	0.45, 0.45, 1.25, 1.25, 0.75	948.79

Improved Marwas, with enhanced geometric regularity and minimized friction losses, facilitated more reliable water delivery, particularly for high-consumption crops like cotton and sugar cane. In contrast, unimproved earthen Marwas exhibited significant flow irregularities, resulting in delayed irrigation and yield penalties, making them unsuitable for high-demand crops such as sugar cane and cotton. Farmers cultivating under unimproved conditions reported up to 20% yield reduction for water-intensive crops, largely due to conveyance losses and poor flow control. These results are consistent with FAO^46^ and Allen et al.^47^ studies, which demonstrated that aligning water delivery infrastructure with crop-specific ETc requirements enhances system efficiency and prevents water stress during critical growth stages. The ability of improved canals to sustain adequate discharge during peak demand months (June–August) aligns with results reported by Gohar and Ward^48^ and Ashour^4^, which found an improvement in irrigation sufficiency following tertiary canal lining in the Nile Delta and stabilized water-use during critical crop growth stages.

This analysis highlights that matching irrigation infrastructure capacity with crop-specific ETc requirements is critical for optimizing water allocation. The improved Marwas supported better scheduling flexibility and more reliable crop water supply, particularly under resource-constrained conditions.

### On-farm flume measurements

To validate on-field irrigation performance, Parshall flume devices were installed at selected Marwas to measure on-farm water application (discharge) during irrigation events^19^. Table 8 presents data from field M3F1, irrigated on February 15, 2024, where sugar beet was cultivated. The recorded flow increased gradually during the initial 15 min, then stabilized reflecting steady irrigation delivery. The total applied water volume was calculated to be 111.37 cubic meters (m^3^). By contrast, previous monitoring of unimproved Marwas revealed discharge variability exceeding 18–25%, due to siltation, uncontrolled flow, and weed obstruction. This discrepancy demonstrates the role of canal rehabilitation in achieving more uniform and measurable flow, supporting equitable and efficient irrigation delivery. This outcome aligns with similar findings from Ashour et al.^4^, which showed that lining or improving tertiary earthen canals reduces discharge variability and increases conveyance efficiency. More stable discharge enhances the predictability of irrigation timing and simplifies scheduling for farmers, supporting more efficient on-farm water management.

**Table 8:** Parshall flume discharge measurements for sugar beet field M3F1 on 15 Feb 2024.

Time	Duration (min)	Upstream (cm)	Downstream (cm)	Discharge Q (m^3^)
Field M3F1 (Sugar Beet)				
08:10	0	10	5	0.00
08:15	15	15	8	11.52
08:20	5	16	10	3.38
08:25	5	17	11	4.02
08:30	5	17	13	4.02
08:35	5	17	13	4.02
08:40	5	17	13	4.02
08:45	5	17	14	4.02
08:50	5	17	14	4.02
08:55	5	17	14	4.02
09:00	5	17	14	4.02
09:05	5	17	14	4.02
09:10	5	17	14	4.02
09:15	5	18	13	4.69
09:20	5	18	13	4.69
09:25	5	18	13	4.69
09:30	5	18	13	4.69
09:35	5	18	13	4.69
09:40	5	18	14	4.69
09:45	5	18	14	4.69
09:50	5	18	14	4.69
09:55	5	18	14	4.69
10:00	5	18	14	4.69
10:05	5	18	14	4.69
10:10	5	18	15	4.69
Total				111.37

### Obtained performance indicators

The obtained performance indicators were analyzed to assess the reliability and consistency of the irrigation performance evaluation. The sensitivity analysis (± 5% variation in discharge and soil-moisture inputs) indicated that the derived indicators (Ea, WUE, WUI) were stable, with deviations below 3%. This indicates the robustness of the field measurements and the stability of the calculated irrigation performance indices.

#### Water application efficiency (Ea)

**Fig. 10:**
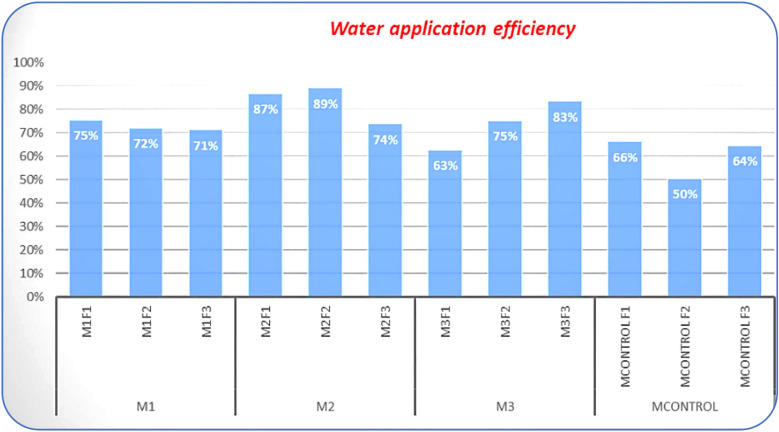
Water application efficiency (Ea) in selected fields of the three improved Marwas compared with the unimproved earthen Marwa.

As illustrated in Fig. 10, the Ea for improved Marwas ranged between 63 and 89%, significantly higher than the 50–66% observed in the unimproved canals. The difference in mean Ea (Δ = 18.5%) was statistically significant at *p* < 0.05, confirming the positive hydraulic effect of canal lining and improved cross-sectional regularity. These improvements are attributed to reduced seepage, minimized operational losses, reduced conveyance losses, smoother canal surfaces (lower Manning’s n ≈ 0.014), minimized seepage and weed resistance, and the introduction of pressurized systems. Conversely, the control fields suffered from water losses through infiltration and non-uniform distribution, particularly toward the tail end of the canals. These results are consistent with benchmarks reported by^49,50^, highlighting the effectiveness of lining in improving water delivery and overall irrigation efficiency. Modernized Marwas with uniform, prismatic cross-sections enhance water delivery efficiency, increase conveyance capacity, reduce friction losses, and inhibit weed growth, particularly critical during peak irrigation months like July and August.

#### Applied water

**Fig. 11:**
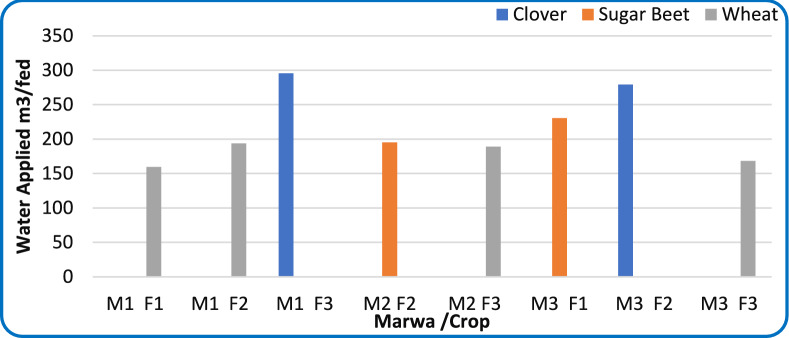
Applied water volumes measured during a single irrigation event (February 2024) for improved and unimproved Marwas.

Figure 11 illustrates the variability in applied water volumes across fields and crop types. Measurements obtained using Parshall Flume revealed significant differences among the sample fields, primarily influenced by crop type and field location along the Mesqa. In improved systems, regulated flow resulted in slightly higher applied water at tail-end fields reflecting controlled distribution. In unimproved Marwas, the opposite trend was observed—tail-end fields experienced reduced flow and frequent water shortages caused by high friction losses, sediment accumulation, and weed obstruction.

Clover recorded the highest applied volumes (≈280–300 m^3^ fed⁻^1^), followed by sugar beet (≈190–230 m^3^ fed⁻^1^), while wheat required less (≈160–190 m^3^ fed⁻^1^). These differences correspond to crop-specific evapotranspiration (ETc) requirements and growth cycles. This indicates that hydraulic improvement enables more efficient and controlled irrigation without compromising productivity. Statistical analysis showed a strong correlation between applied water and crop water requirements in improved Marwas (R^2^ = 0.82), compared with weaker alignment in earthen systems (R^2^ = 0.48). The coefficient of variation (CV) further confirmed this distinction—6.8% for improved fields versus 17.3% for unimproved ones—indicating greater uniformity in modernized canals.

Similar results were documented by Ragab et al.^51^, who found that improvements to tertiary canals in the Nile Delta enhanced irrigation efficiency, reducing the volume of applied water while maintaining or even increasing crop yields. Likewise, the FAO^52^ emphasized that interventions such as canal lining and hydraulic control substantially reduce conveyance losses caused by seepage and tail-end overflow, thereby improving the overall performance and equity of irrigation systems.

#### Water use efficiency

**Fig. 12:**
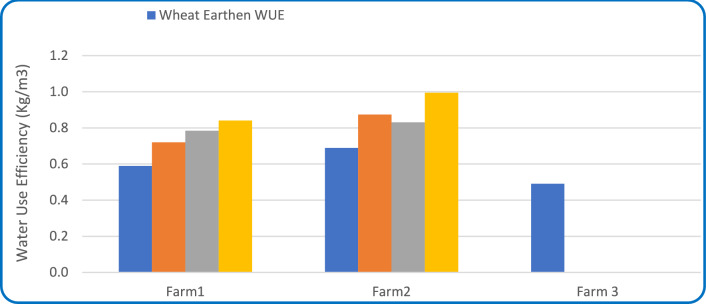
Water use efficiency (WUE) in earthen (unimproved) and improved Marwas during the winter season of 2024.

As shown in Fig. 12, WUE in improved Marwas ranged from 0.72 to 0.87 (equivalent to 72–87%), whereas the unimproved canals recorded lower values ranging from 0.59 to 0.69 (59–69%). Sugar beet and wheat exhibited the best WUE due to efficient water uptake and deep root systems. The improved systems achieved not only higher water productivity but also more consistent field-level outcomes, as confirmed by a coefficient of determination (R^2^ = 0.91, *P* < 0.05) between WUE and Ea values, indicating that application efficiency strongly influences use efficiency. These results are consistent with Elkamhawy et al.^38^ who reported that canal lining and rehabilitation in the Nile region significantly reduced seepage losses and enhanced conveyance efficiency, and Ahmed^53^ found that adopting modern irrigation systems, such as drip irrigation in Fayoum, reduced applied water by 17–27%, thereby improving overall WUE.

#### Water use index (WUI)

**Fig. 13:**
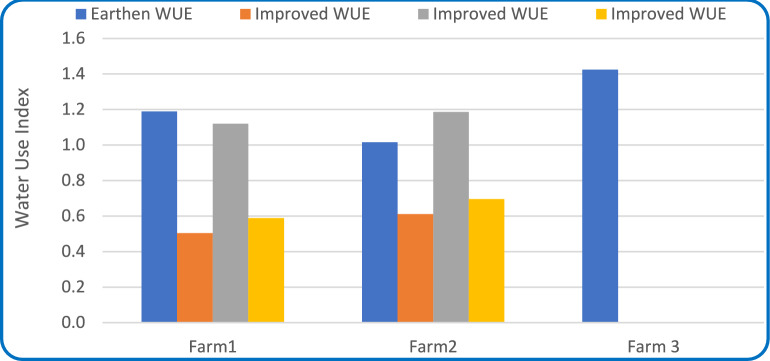
Water use index (WUI) for of unimproved (earthen) and improved Marwas during the winter season in 2024.

As illustrated in Fig. 13, Improved Marwas recorded WUI values between 0.5 and 1.2, whereas unimproved ones showed 1.2–1.4, indicating frequent over-irrigation. A WUI value near 1.0 denotes optimal irrigation alignment. The distribution of WUI among improved systems followed a normal distribution (Shapiro–Wilk *p* = 0.22), suggesting controlled water application, while unimproved systems showed a skewed distribution (*p* < 0.05), consistent with uncontrolled irrigation behavior. This confirms that improved Marwas promoted rational, controlled irrigation,

Lower water use index (WUI) values in improved systems indicate more efficient and rational irrigation, with applications closely matching crop needs. These gains result from canal lining and controlled irrigation, which enhance water delivery precision and reduce excess use^5,54^. Some lower WUI values reflected early irrigation during low-demand stages, emphasizing the need for improved scheduling and soil moisture monitoring^55^. In contrast, higher WUI values in unimproved Marwa indicate over-irrigation due to poor control and traditional flooding. Overall, Marwa rehabilitation enhances conveyance efficiency and promotes equitable, sustainable water use^54^.

#### Water distribution equity

**Fig. 14:**
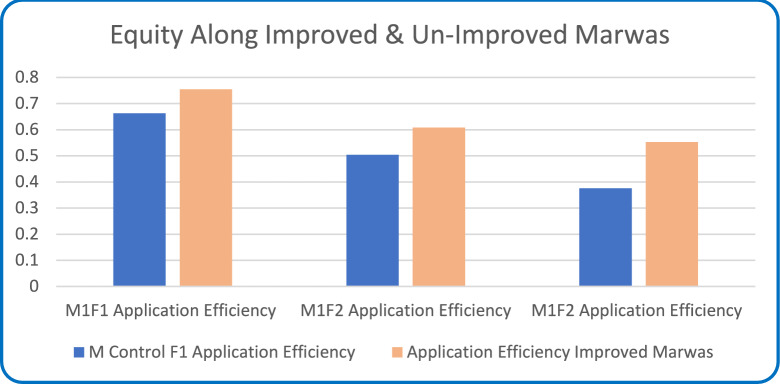
Water distribution equity index along improved and unimproved Marwas.

Water distribution, Fig. 14, revealed that improved Marwas achieved an index of 0.93 compared with 0.68 for unimproved canals. This improvement reflects the hydraulic benefits of canal lining and regulated irrigation, which standardized canal geometry, minimized head losses, and stabilized discharge along the canal length. Field surveys also reported about a 40% reduction in irrigation delays for tail-end users under rehabilitated systems, confirming that infrastructure upgrades resulted in more reliable and equitable water delivery.

Beyond hydraulic gains, these improvements have significant social implications. Equitable access to irrigation water fosters cooperation among farmers, reduces competition and conflict, and supports collective irrigation scheduling. These findings illustrate that tertiary-level rehabilitation enhances both the technical efficiency and the social sustainability of irrigation systems^36,54^.

#### Water productivity

Table 9 provides data that contrasts fields served by improved Marwas with modern irrigation systems and those supplied by unimproved Marwas using traditional surface irrigation systems. The results show a clear difference in water efficiency and crop yield between the two approaches. Improved Marwas applied 2130 m^3^/Fed and produced 3710 kg/Fed of grain yield, achieving a water productivity of 1.75 kg/m^3^. In contrast, unimproved Marwas applied 2830 m^3^/Fed and yielded only 2650 kg/Fed, with a water productivity of 0.93 kg/m^3^. This productivity boost reflects better irrigation control, conveyance efficiency, reduced seepage and application losses, more precise water application, and higher yields, particularly for wheat and sugar beet crops. Similar findings were reported by Ashour et al.^37^, who documented better hydraulic performance in lined tertiary canals in Kafr El-Sheikh.

**Table 9:** Mean applied irrigation, grain yield and water productivity for wheat in fields served by improved and unimproved Marwas during the 2024 winter season.

	Applied irrigation (m^3^/Fed)	Grain yield (Kg/Fed)	Water productivity (Ton/m^3^)
Fields served by improved Marwas and modern irrigation systems	2130	3720	1.75
Fields served by unimproved Marwas and traditional surface irrigation systems	2830	2650	0.93

While canal lining significantly enhances hydraulic performance, it can also reduce groundwater recharge. Elkamhawy et al.^38^ quantified seepage reduction efficiencies of 99% for concrete liners, 96% for geo-membrane liners, and 54% for bentonite liners along the Ismailia Canal. However, cracked or deteriorated liners reduced efficiency to as low as 25%, and when combined with aquifer pumping, to 16%. These results highlight the importance of regular inspection and maintenance to sustain hydraulic performance. Periodic desilting, structural repair, and vegetation control are therefore essential to maintain long-term functionality.

Nevertheless, potential long-term challenges remain. While canal lining improves short-term efficiency, it may also reduce groundwater recharge, as shown in modelling for the Nile Delta, where lining reduced canal–aquifer leakage to ~ 10% of the unlined scenario^38^. This suggests that integrated water resource management (IWRM) will be necessary to ensure long-term hydrological sustainability.

Furthermore, maintenance challenges such as siltation, concrete cracking, and vegetation regrowth could gradually diminish efficiency over time. Routine desilting and periodic lining inspection are therefore recommended.

### Socioeconomic assessment

A socioeconomic assessment was conducted based on questionnaire responses collected from 20% of all farmers within the study command area, comprising 20 beneficiaries and 20 non-beneficiaries of the Marwa rehabilitation project. The survey focused on several key aspects, including time and cost savings, water quality, weed control in Marwas, improved water regulation, the ability to cultivate new crops, and increased irrigation efficiency. The evaluation approach was aligned with FAO’s participatory irrigation assessment framework, emphasizing farmer involvement in performance monitoring^56^.

Although the sample size offers valuable insights, its restriction to a single district may introduce local bias due to local socioeconomic or environmental conditions. Additionally, external factors such as market access, weather variability, and farm size may have influenced responses. Future studies should therefore expand to multi-district sampling to improve representativeness.

To evaluate differences in farmers’ perceptions and acceptance levels between beneficiaries and non-beneficiaries, a one-way Analysis of Variance (ANOVA) was applied to the survey data. Assumptions of normality and homoscedasticity were verified using the Shapiro–Wilk and Levene tests. Results indicated statistically significant differences (*p* < 0.05) in perceived irrigation efficiency, weed reduction, water quality, and time savings, with beneficiaries consistently reporting higher satisfaction.

**Fig. 15:**
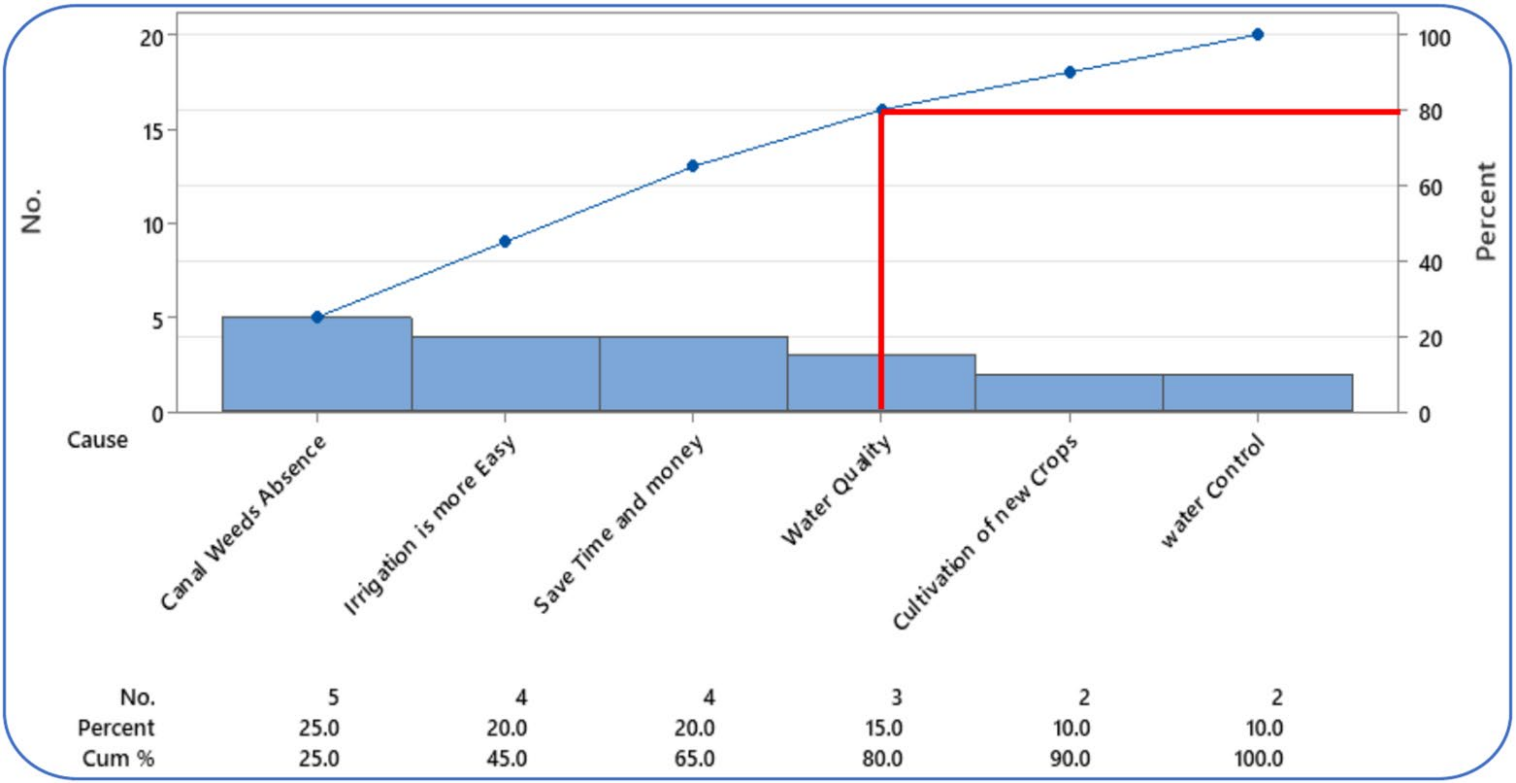
Pareto chart-farmer’s preference to implement the Marwa improvement system.

A Pareto chart (Fig. 15) was used to identify farmers’ key preferences for implementing the Marwa improvement system, highlighting the most frequently cited benefits that motivated adoption. The main reasons reported were the absence of canal weeds (25%), ease of irrigation (20%), saving time and money (20%), and improved water quality (15%), collectively representing 80% of all responses. The results indicate that these four factors are the most influential in motivating farmers to adopt the system. Over 80% of respondents supported the rehabilitation project, citing reduced time and money spent on irrigation, improved water control, elimination of aquatic weeds, and enhanced crop productivity. The remaining factors (cultivation of new crops (10%) and better water control (10%)) contribute comparatively less influential. Overall, the figure highlights that improving canal management, simplifying irrigation, and enhancing efficiency are the key aspects driving farmers’ interest in the Marwa improvement system.

**Fig. 16:**
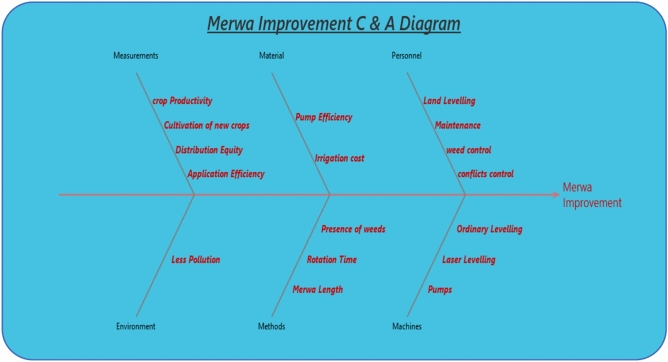
Ishikawa chart illustrating the effect and cause diagram of Marwa improvement.

Further analysis using the Ishikawa (Cause-effect) diagram (Fig. 16) identified the primary factors contributing to the successful implementation of the Marwa improvement system. The most prominent causes included land levelling, routine maintenance of Marwas and pumps, effective weed control, and a reduction in water-related conflicts resulting from improved water distribution equity, especially at the canal tail ends. Farmers also reported visible improvements such as the suppression of weed growth, which otherwise hindered water flow, and the prevention of waterlogging in low-lying areas adjacent to the Marwas. These improvements not only enhanced system efficiency but also minimized environmental pollution and promoted more sustainable water management practices.

Economic and Spatial Impacts: In addition to agronomic benefits, the rehabilitation also yielded spatial advantages and infrastructural advantages. The lining of Marwas allowed previously unutilized portions of canal cross-sections to be repurposed for crop cultivation or conversion into access roads, facilitating the transport of agricultural inputs and market access. Questionnaire results confirmed a substantial increase in crop yields and higher household income, which farmers attributed to better irrigation control, shorter irrigation times, and reduced water wastage. These combined technical and economic gains collectively explain the high level of farmer satisfaction with the project. Farmers also expressed a strong willingness to adopt more advanced irrigation technologies, suggesting a favorable outlook for future integration of smart irrigation tools.

However, achieving equitable water distribution remains a critical prerequisite for standardizing irrigation schedules. Without fairness across the entire length of the Marwa, downstream users may still face challenges, limiting the effectiveness of automation and scheduling efforts. Moreover, while canal lining significantly enhances water delivery efficiency and supports higher productivity, it may also reduce seepage and, consequently, groundwater recharge. This introduces a potential trade-off between surface water efficiency and subsurface water sustainability. Integrated water resources management (IWRM) strategies will therefore be necessary to ensure system balance, long-term agricultural water use, securing both current productivity and future water availability.

The findings suggest that similar Marwa rehabilitation projects could be successfully replicated in other districts with comparable agronomic and socioeconomic conditions, provided that maintenance is ensured, water equity is upheld, and farmers remain actively engaged in system monitoring and management.

### Integrated evaluation under the IPEF framework

The Integrated Performance Evaluation Framework (IPEF) was employed to synthesize hydraulic, agronomic, and socioeconomic indicators to assess tertiary canal rehabilitation^33,57^. Results revealed a moderately significant positive correlation between technical improvements (application efficiency, water distribution uniformity, and water productivity) and farmer satisfaction (r = 0.68, *p* < 0.01).

The integrated assessment of field measurements and farmer feedback suggests that tertiary-level canal rehabilitation improved conveyance, distribution uniformity, and irrigation efficiency, while aligning with user preferences. These outcomes are consistent with FAO frameworks for water productivity and yield-gap assessment^57^ and suggest alignment with MWRI- and FAO-supported irrigation modernization programs in Egypt^58^.

However, several methodological constraints should be acknowledged. The IPEF analysis was limited by the relatively small sample size and the partial availability of some socioeconomic indicators, both of which may affect the strength and generalizability of multi-dimensional correlations. Furthermore, the cross-sectional design provides only a snapshot of system performance, limiting the ability to conclude long-term behavioral adaptation or sustained productivity outcomes.

## Conclusion

The geometric and hydraulic analyses clearly confirm that rehabilitated Marwas exhibit uniform, prismatic cross-sections that significantly enhance conveyance capacity and hydraulic efficiency compared with the irregular, friction-prone channels of unimproved systems. These improvements minimize head losses, reduce seepage, suppress weed growth, and prevent localized waterlogging, thereby contributing to more consistent and equitable water distribution along the tertiary network. As a result, application efficiency reached 89% in rehabilitated systems, consistent with measured field performance.

From a socioeconomic perspective, farmers reported substantial reductions in irrigation time and fuel consumption, along with moderate but measurable increases in crop, and a substantial decline in water-related disputes. These findings highlight the positive contribution of Marwas’ rehabilitation to community relations, economic resilience, and social equity. Additional spatial benefits, such as converting canal margins into cultivated strips or access routes, were also observed but remain context-specific.

The IPEF was applied using the indicators that were most relevant to the study objectives and for which reliable field data were available. While not all components of the full framework could be evaluated, the selected indicators provided a robust basis for assessing the hydraulic and socioeconomic impacts of Marwa rehabilitation within the context of the available dataset. This study provides evidence-based recommendations for improving tertiary-level irrigation systems through group-based irrigation scheduling and the adoption of site-specific irrigation technologies such as soil-moisture monitoring tools. Farmer training on irrigation scheduling, soil–water balance, and system maintenance will be essential for sustaining outcomes. These actions are broadly aligned with national water-management strategies.

Nevertheless, certain limitations warrant attention. The study’s results are limited to a single geographic location and a small sample size, which may constrain the generalizability of the findings. Socioeconomic data relied partly on self-reported farmer responses, which may introduce recall or response bias. Moreover, reduced canal seepage associated with rehabilitation may influence local groundwater recharge, requiring integrated monitoring. Similarly, initial rehabilitation costs may also limit adoption by smallholders unless supported by microfinance, microcredit schemes, or a cost-sharing mechanism.

This study offers a replicable model for evaluating tertiary-canal rehabilitation in arid and semi-arid regions, though broader multi-season and multi-location assessments are needed to validate scalability.

In conclusion, Marwa’s rehabilitation in Menia Governorate has demonstrated significant measurable gains in water-use efficiency, distribution equity, and agricultural productivity. Future interventions should adopt a holistic framework that links physical infrastructure upgrades with capacity development, digital monitoring tools, and participatory water-governance mechanisms to ensure long-term sustainability and scalability.

## Supplementary Information

Supplementary Information.

## Data Availability

All data generated or analyzed during this study are included in this published article. Additional datasets generated and analyzed during the current study are available from the corresponding author on reasonable request. No external repositories were used.
