# Supplementary material for: Ex post impact assessment of Marwa rehabilitation on irrigation performance under water scarcity in Egypt
Source: Sci Rep. 2026 Jan 20;16:2636. doi: 10.1038/s41598-025-34200-2 (PMC12823578; doi:10.1038/s41598-025-34200-2)
Supplement: Supplementary file 1 — Supplementary Information. [file 41598_2025_34200_MOESM1_ESM.pdf]

Appendix I. Farmer Survey Questionnaire

This questionnaire was administered to a representative group of farmers (n = 40 [20% of total farmers]) in the Hafez El Sharkeya Canal command area, Menia Governorate. The questionnaire was used to complement technical measurements by providing qualitative and quantitative insights into farmer behavior, preferences, and challenges.

Section 1: General Information

1. Study Location:
   - Governorate: ____________
   - Village: ____________
   - District/Center: ____________
   - Area: ____________
2. Form Recorder Details:
   - Name: ____________
   - Phone: ____________
   - Job/Occupation: ____________
3. Farmer Name: ____________
4. Date: ____________
5. Farm Type/Ownership: ____________
6. Owner Name: ____________
7. Start of Using Agricultural Machines: ____________
8. Farm Size (feddan or hectare): ____________

Section 2: Irrigation System Details

9. Irrigation Channel Name: ____________
10. Water Source Used:

☐ Modern lined channel with pipes ☐ Modern lined channel

☐ Earthen channel ☐ Groundwater / mixed wastewater

11. Your Position on the Irrigation Channel: ☐ Beginning ☐ Middle ☐ End
12. Name of the Water Distributor (Marwa):

☐ Modern lined Marwa with pipes ☐ Lined Marwa ☐ Earthen Marwa

13. Your Position on the Marwa: ☐ Beginning ☐ Middle ☐ End
14. Irrigation Method Used on Your Land:

☐ Flooding in lines ☐ Flooding in basins ☐ Modern irrigation system (sprinkler / drip) ☐ Developed irrigation

15. Irrigation Cost per Feddan (e.g., pump operation): ___________ Other costs (if any): ___________
16. Water Quality:

☐ Excellent ☐ Good

17. Shift Duration:
    - Work days: ___________
    - Idle days: ___________
18. Do you experience delays in shift timing? ☐ Yes ☐ No

Section 3: Water Management and Practices

19. Is water sufficient during your shift? ☐ Yes ☐ No
20. Which fields suffer the most from water shortage and why? ___________

21. Were there water access problems in the fields, and how did these improve after the project?
☐ Problems existed and improved
☐ Problems existed and worsened
☐ No problems, new issues emerged Describe problems (if any): ___________
22. Is water availability now more, less, or the same compared to before the development? ☐ Less ☐ More ☐ Same
23. Do you prefer the developed irrigation or earthen channels?
☐ Development is better ☐ Earthen channels Why? ___________
24. Have water disputes with neighbors decreased?
☐ Yes, decreased ☐ No, not decreased ☐ Same
25. Does water reach the ends more efficiently than before?
☐ Yes ☐ No ☐ Same
26. Did you benefit from the development project?
☐ Yes ☐ No If no, would you like your irrigation channel to be developed?
☐ Yes ☐ No
27. Do you have low spots in your farm causing water accumulation? ☐ Yes ☐ No
28. How do you level your land and what do you use? ___________
29. Have you leveled your land using laser technology? ☐ Yes ☐ No
30. How many times have you leveled your land? ___________
31. When was the last time you leveled your land with a laser? (Year/Month) ___________
32. Did you notice any change in irrigation after leveling with laser compared to other methods? ☐ Yes ☐ No
33. Are there weeds in the channels after development? ☐ Yes ☐ No
34. Do you participate in cleaning the channel / irrigation canal / drains in the village? ☐ Always ☐ Sometimes ☐ Rarely ☐ No
35. Does the association operate / maintain / clean the channel / irrigation regularly?
☐ Always ☐ Sometimes ☐ Rarely ☐ No
36. When was the last cleaning of the Marwa / channel / drain? ___________
37. Does the irrigation pump work efficiently?
☐ Yes ☐ No If yes, what is its hourly cost? ___________ Is maintenance/fuel easily available? ☐ Yes ☐ No

Section 4: Economic and Production Impacts

38. Has your net income increased? ☐ Yes ☐ No ☐ Decreased
39. Did development result in higher quality crops or higher productivity?
☐ Higher quality crop ☐ Not higher quality ☐ Higher productivity ☐ Lower productivity
40. Have new crop varieties been introduced that were previously unavailable?
☐ Yes, new varieties introduced ☐ No
41. Would you recommend expanding the modern irrigation development project?☐ Yes ☐ No Why? ___________
42. What support do you need from authorities to sustain the system?
☐ Maintenance ☐ Training ☐ Equipment ☐ Financial ☐ Organizational

Ethical Note

Participation in the survey was voluntary. All responses were collected and used solely for academic purposes. Farmers provided informed consent before completing the questionnaire.
